# The significance of cytogenetics for the study of karyotype evolution and taxonomy of water bugs (Heteroptera, Belostomatidae) native to Argentina

**DOI:** 10.3897/CompCytogen.v7i2.4462

**Published:** 2013-04-29

**Authors:** Chirino Mónica Gabriela, Alba Graciela Papeschi, María José Bressa

**Affiliations:** 1 Instituto de Ecología, Genética y Evolución de Buenos Aires, Departamento de Ecología, Genética y Evolución, Facultad de Ciencias Exactas y Naturales, Universidad de Buenos Aires, Ciudad Universitaria, Pabellón II, C1428EHA, Ciudad Autónoma de Buenos Aires, Argentina; 2 Laboratorio de Entomología Aplicada y Forense, Departamento de Ciencia y Tecnología, Universidad Nacional de Quilmes, Roque Sáenz Peña 352, B1876BXD, Bernal, Buenos Aires, Argentina

**Keywords:** Heteroptera, holokinetic chromosomes, karyotype evolution mechanisms, multiple sex chromosomes, rDNA-FISH

## Abstract

Male meiosis behaviour and heterochromatin characterization of three big water bug species were studied. *Belostoma dentatum* (Mayr, 1863), *Belostoma elongatum* Montandon, 1908 and *Belostoma gestroi* Montandon, 1903 possess 2n = 26 + X_1_X_2_Y (male). In these species, male meiosis is similar to that previously observed in *Belostoma* Latreille, 1807. In general, autosomal bivalents show a single chiasma terminally located and divide reductionally at anaphase I. On the other hand, sex chromosomes are achiasmatic, behave as univalents and segregate their chromatids equationally at anaphase I. The analysis of heterochromatin distribution and composition revealed a C-positive block at the terminal region of all autosomes in *Belostoma dentatum*, a C-positive block at the terminal region and C-positive interstitial dots on all autosomes in *Belostoma elongatum*, and a little C-positive band at the terminal region of autosomes in *Belostoma gestroi*. A C-positive band on one bivalent was DAPI negative/CMA_3_ positive in the three species. The CMA_3_-bright band, enriched in GC base pairs, was coincident with a NOR detected by FISH. The results obtained support the hypothesis that all species of *Belostoma* with multiple sex chromosome systems preserve NORs in autosomal bivalents. The karyotype analyses allow the cytogenetic characterization and identification of these species belonging to a difficult taxonomic group. Besides, the cytogenetic characterization will be useful in discussions about evolutionary trends of the genome organization and karyotype evolution in this genus.

## Introduction

Belostomatidae include some of the largest heteropteran species, which are general predators that play an important role as biological agents in aquatic environments (Menke 1979, Smith 1997, Saha et al. 2010). This family has a cosmopolitan distribution in tropical and subtropical areas in the world (Schnack 1976, Polhemus and Polhemus 2008). In South America, the genus *Belostoma* Latreille, 1807 is the most diverse and includes 61 species mainly distributed from Colombia and Brazil to Argentina and Chile (Heckman 2011). Nevertheless, cytogenetic reports in *Belostoma* from South America comprise the male chromosome complement of 15 species and male meiosis analysis of 13 species (Table 1). Ten of these species show a modal diploid chromosome number 2n = 29 = 26 + X_1_X_2_Y (male) and five species possess reduced chromosome numbers and a simple sex chromosome system XY/XX (male/female) (Table 1).

All species of *Belostoma* analyzed possess holokinetic chromosomes, i.e. chromosomes without a primary constriction and therefore without a localized centromere. Autosomal bivalents are synaptic and chiasmatic, whereas sex chromosomes are asynaptic and achiasmatic, and behave as univalents in first male meiotic division. However, at metaphase II sex chromosomes associate end-to-end through the so called touch-and-go pairing, forming a pseudo-bivalent or pseudo-multivalent. In the first meiotic division, autosomal bivalents segregate reductionally while sex chromosomes divide equationally (Ueshima 1979, Papeschi and Bidau 1985, Suja et al. 2000, Papeschi and Bressa 2006, Bardella et al. 2012). During meiosis, the kinetic activity is restricted to the chromosome ends and the chromosomes can be regarded as telokinetic (Motzko and Ruthmann 1984).

Most hypotheses on karyotype evolution in Heteroptera include both autosomal and sex chromosome fusions and fragmentations (Ueshima 1979, Manna 1984, Thomas 1987, Papeschi 1994, 1996, Pérez et al. 2004). The cytogenetic data available for *Belostoma* allow to hypothesize that current karyotypes with a multiple sex chromosome system (X_n_Y/X_n_X_n_, male/female) are derived through fragmentation of the ancestral X from an XY sex chromosome system. On the other hand, reduced chromosome complements with simple sex chromosome system (XY/XX, male/female) have probably originated through several chromosomal fusions (Papeschi 1996, Papeschi and Bressa 2006, Bardella et al. 2012).

The aim of this study was to perform a detailed comparison of male meiosis behaviour and examine the structure of the holokinetic chromosomes by means of C- and fluorescent bandings, and fluorescent *in situ* hybridization (FISH) with 18S rDNA probes in *Belostoma dentatum* (Mayr, 1863), *Belostoma elongatum* Montandon, 1908 and *Belostoma gestroi* Montandon, 1903. The female complement and the male meiosis of *Belostoma elongatum* and *Belostoma gestroi* are described for the first time. These results allowed us to distinguish morphologically similar species and, also, led us to propose a scenario of karyotype evolution in the genus *Belostoma*.

**Table 1.** Diploid chromosome number, chromosome bandings and nucleolar organizer region (NOR) detected by FISH in South American *Belostoma* species. *A: autosomal bivalent, **X, Y: sex chromosomes.

Species	2n (male)	C bands	DAPI/CMA_3_ bands	rDNA by FISH	References
*Belostoma bergi* (Montandon), 1899	26 + X_1_X_2_Y	no	no	--	Papeschi and Bressa 2004
*Belostoma bifoveolatum* Spinola, 1852	26 + X_1_X_2_Y	yes	yes	--	Papeschi 1991, Chirino and Bressa 2011
*Belostoma candidulum* Montandon, 1903	14 + XY	yes	yes	--	Bardella et al. 2012
*Belostoma cummingsi* De Carlo, 1935	26 + X_1_X_2_Y	no	no	--	Papeschi and Bidau 1985
*Belostoma dentatum* (Mayr, 1863)	26 + X_1_X_2_Y	yes	yes	A^*^	Papeschi and Bidau 1985, Papeschi 1991, this study
*Belostoma dilatatum* (Dufour, 1863)	26 + X_1_X_2_Y	yes	no	--	Papeschi 1992
	26 + X_1_X_2_ X_3_Y	yes	yes	--	Bardella et al. 2012
*Belostoma discretum* Montandon, 1903	26 + X_1_X_2_Y	yes	yes	--	Papeschi 1992, Chirino and Bressa 2011
*Belostoma elegans* (Mayr, 1871)	26 + X_1_X_2_Y	yes	yes	A^*^	Papeschi 1988, 1991, Papeschi and Bidau 1985,
					Papeschi and Bressa 2006
*Belostoma elongatum* Montandon, 1908	26 + X_1_X_2_Y	yes	yes	A^*^	Papeschi and Bressa 2006, this study
*Belostoma gestroi* Montandon, 1903	26 + X_1_X_2_Y	yes	yes	A^*^	Papeschi and Bressa 2006, this study
*Belostoma martini* (Montandon, 1899)	26 + X_1_X_2_Y	yes	no	--	Papeschi 1991
*Belostoma micantulum* (Stål, 1860)	14 + XY	yes	yes	X,Y^*^	Papeschi 1988, Papeschi and Bressa 2006
*Belostoma orbiculatum* Estévez & Polhemus, 2001	14 + XY	yes	yes	--	Papeschi 1996, Chirino and Bressa 2011
	14 + X_1_X_2_Y				Papeschi 1996
*Belostoma oxyurum* (Dufour, 1863)	6 + XY	yes	yes	X,Y^**^	Papeschi 1988, 1995, Papeschi and Bressa 2006
*Belostoma plebejum* (Stål, 1858)	14 + XY	no	no	--	
	13 + XY				Papeschi 1994
	14 + X_1_X_2_Y				

## Material and methods

### Insects

For meiotic analysis, adults and nymphs of *Belostoma elongatum* (9 males and 8 females) and *Belostoma gestroi* (4 males and 12 females) were collected from 1988 to 1990 in several fields from Buenos Aires, Santa Fe, Entre Ríos, Corrientes and Misiones provinces, all in Argentina (Table 2). For chromosome bandings and fluorescent *in situ* hybridization (FISH) technique, adults of *Belostoma dentatum* (3 males and 1 female), *Belostoma elongatum* (3 males) and *Belostoma gestroi* (2 males and 1 female) were collected from 2010 to 2011 in Corrientes province (Argentina) (Table 2). Collected adults were identified according to the keys provided by Schnack (1976) and Heckman (2011).

**Table 2.** Species, provenience, geographical coordinates, and number of adults’ collected and examined of *Belostoma* for chromosomal analyses discriminated by gender.<br/>

**Species**	**Chromosomal analyses**	**Localities from Argentina**	**Coordinates**	**N° of individuals**
*Belostoma dentatum*	C- and DAPI-CMA_3_ bandings	San Pedro, Buenos Aires	33°40'33"S, 59°39'47"W	3 males
	FISH technique	Corrientes, Corrientes	27°28'16"S, 58°50'22"W	1 female
*Belostoma elongatum*	Chromosome complement	Arroyo Cuay Grande, Corrientes	28°28'16"S, 58°50'22"W	1 female
	Male meiotic behaviour	Lagos de Stieler, Misiones	26°34'2"S, 54°45'57"W	1 male
		Valle Hermoso, Misiones	26°23'10"S, 54°27'58"W	8 males, 7 females
	C- and DAPI-CMA_3_ bandings FISH technique	Corrientes, Corrientes	27°28'16"S, 58°50'22"W	3 males
*Belostoma gestroi*	Chromosome complement	Río San Pedro, Buenos Aires	33°40'33"S, 59°39'47"W	1 male
	Male meiotic behaviour	Rincón Norte, Santa Fe	31°36'4"S, 60°34'12"W	3 males, 11 females
		Santa Rosa, Santa Fe	31°26'00"S, 60°22'00"W	1 female
	C- and DAPI-CMA_3_ bandingsFISH technique	Corrientes, Corrientes	27°28'16"S, 58°50'22"W	2 males, 1 female

### Chromosome preparations

The captured specimenswere brought alive to the laboratory and reared until their gonads were dissected out. For meiotic analysis, the adults and nymphs were fixed for 15–30 min in freshly prepared fixative (ethanol:glacial acetic acid, 3:1). Afterwards, gonads were dissected out and kept at 4° C in 70% ethanol. Slides were prepared by the squash technique in a drop of 2% iron-propionic haematoxylin following conventional procedures (Sáez 1960). For C- and fluorescent bandings, and FISH technique, gonads were dissected in a physiological saline solution for *Ephestia* Guenée, 1845 (Glaser 1917, cited by Lockwood 1961), swollen for 15 min in a hypotonic solution (0.075 M KCl), and fixed for 15-30 min in freshly prepared Carnoy fixative (ethanol:chloroform:glacial acetic acid, 6:3:1). Spread chromosome preparations were made in a drop of 60% acetic acid with the help of tungsten needles and spread on the slide using a heating plate at 45° C as described in Traut (1976). The preparations were dehydrated in an ethanol series (70, 80 and 96%, 30 sec each) and stored at -20º C until use.

### Chromosome bandings

Heterochromatin content, distribution and nucleotide composition were analysed by means of C- and sequential fluorescent DAPI and CMA_3_ bandings. C-banding was performed according to Papeschi (1988), and the pre-treated slides were stained with 4’6-diamidino-2-phenylindole (DAPI; Fluka BioChemika, Sigma Aldrich Production GmbH, Buchs, Switzerland) for a better resolution of C-bands (Poggio et al. 2011). Fluorescent banding with AT-specific DAPI and GC-specific chromomycin A_3_ (CMA_3_; Fluka BioChemika) was carried out following Poggio et al. (2011).

### Fluorescence *in situ* hybridization

Unlabelled 18S ribosomal DNA (rDNA) probes were generated by polymerase chain reaction (PCR) using universal arthropod primers: forward 5´-CCTGAGAAACGGCTACCACATC-3´ and reverse 5´-GAGTCTCGTTCGTTATCGGA-3´ (Whiting 2002). Total genomic DNA of *Dysdercus albofasciatus* Berg, 1878, obtained by standard phenol-chloroform-isoamylalcohol extraction, was used as a template. PCR was done following the procedure described in Fuková et al. (2005). The PCR product showed a single band of about 1,000 bp on a 1% agarose gel. The band was recovered from the gel and purified by using a QIAquick Gel Extraction Kit (Quiagen GmbH, Hilden, Germany). The 18S rDNA fragment was re-amplified by PCR and then labeled with biotin 14-dATP by nick translation using a BioNick Labeling System (Invitrogen, Life Technologies Inc., San Diego, CA, USA). FISH with a biotinylated 18S rDNA probe was carried out following the procedure in Sahara et al. (1999) with several modifications described by Fuková et al. (2005) and Bressa et al. (2009).

### C-positive heterochromatin and DNA measurements

Data of C-positive heterochromatin percentage and the haploid DNA content in *Belostoma dentatum*, *Belostoma elongatum* and *Belostoma gestroi* are part of the results obtained by Papeschi in her Ph.D. Thesis (1992). The procedures of C-positive heterochromatin percentage and the haploid DNA content were published by Papeschi in 1991 and 1988, respectively. Hence, these results were mentioned only in this paper to analyze and discuss the extent of karyotype uniformity in these three species. Briefly, the study of the C-positive heterochromatin measurements was performed on meiotic cells. For each species at least 10 C-banded cells at diakinesis and without superimposed bivalents were photographed. The percentage of C-positive heterochromatin was calculated as the C-positive area divided by the total chromosome area. The DNA content was carried out by Feulgen microdensitometry (Papeschi 1988) in individuals fixed similar time ago (1–3 months) (Papeschi 1991).

### Statistical analysis

The total chromosome length measurements (TCL) were performed with Micro Measure for Windows, version 3.3. The TCL of all bivalents and sex chromosomes were performed in metaphase I. Differences in TCL among species were compared by using one-way analysis of variance (ANOVA), with Fisher adjusted a posterior contrast. Statistical analyses were done using Statview software (SAS Institute Inc., 1992-1998).

### Microscopy, photographs and image processing

Preparations were observed in epifluorescence microscopes: Zeiss Laborlux (Carl Zeiss, Germany) equipped with an analogue camera and Leica DMLB equipped with a Leica DFC350 FX CCD camera and Leica IM50 software, version 4.0 (Leica Microsystems Imaging Solutions Ltd., Cambridge, UK). Photomicrographs from meiotic chromosome preparations were taken using Kodak colour Supra print film 400 ASA. Black-and-white images of chromosomes from C- and fluorescent bandings and FISH technique were recorded separately for each fluorescent dye with the CCD camera. Images were pseudo-coloured (light blue for DAPI, green for CMA_3_, and red for Cy3), and processed with an appropriate software.

## Results

### Male chromosome complement and meiosis

**Figure 1.**
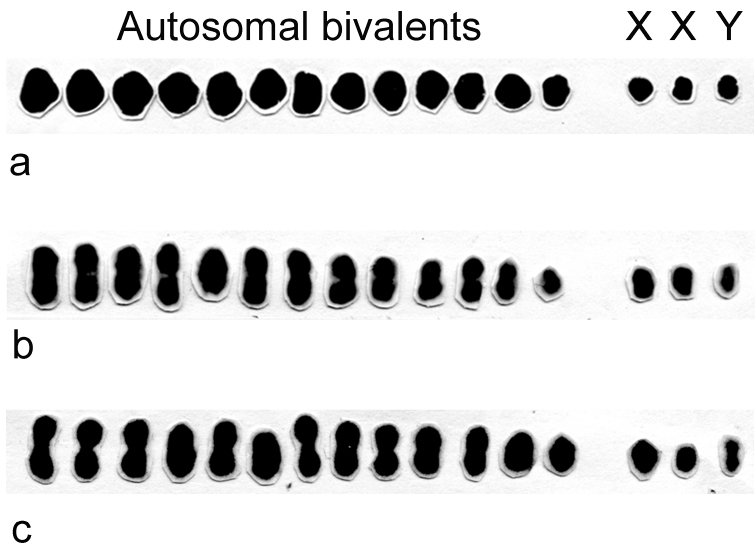
Male meiotic karyotypes of *Belostoma dentatum* (**a**), *Belostoma elongatum* (**b**) and *Belostoma gestroi* (**c**), 2n = 13II + X_1_X_2_Y, stained with 2% iron-propionic haematoxylin.

Male meiotic karyotypes based on metaphase I autosomal bivalents (II) and sex univalents of *Belostoma dentatum*, *Belostoma elongatum* and *Belostoma gestroi* show a male diploid chromosome number 2n = 13II + X_1_X_2_Y (Fig. 1). In the three species, the autosomes decrease gradually in size, both X chromosomes differ slightly in size and the Y chromosome is the smallest of the complement. The chromosome complement and male meiotic behaviour of *Belostoma dentatum* have already been described (Papeschi and Bidau 1985). The three species of *Belostoma* show statistical differences in total chromosome length (TCL) (F _2, 93_ = 8.484; P = 0.0004), which is higher in *Belostoma dentatum* (39.43 ± 3.72 µm), intermediate in *Belostoma elongatum* (37.03 ± 2.96 µm) and lower in *Belostoma gestroi* (33.31 ± 3.64 µm).

**Figure 2.**
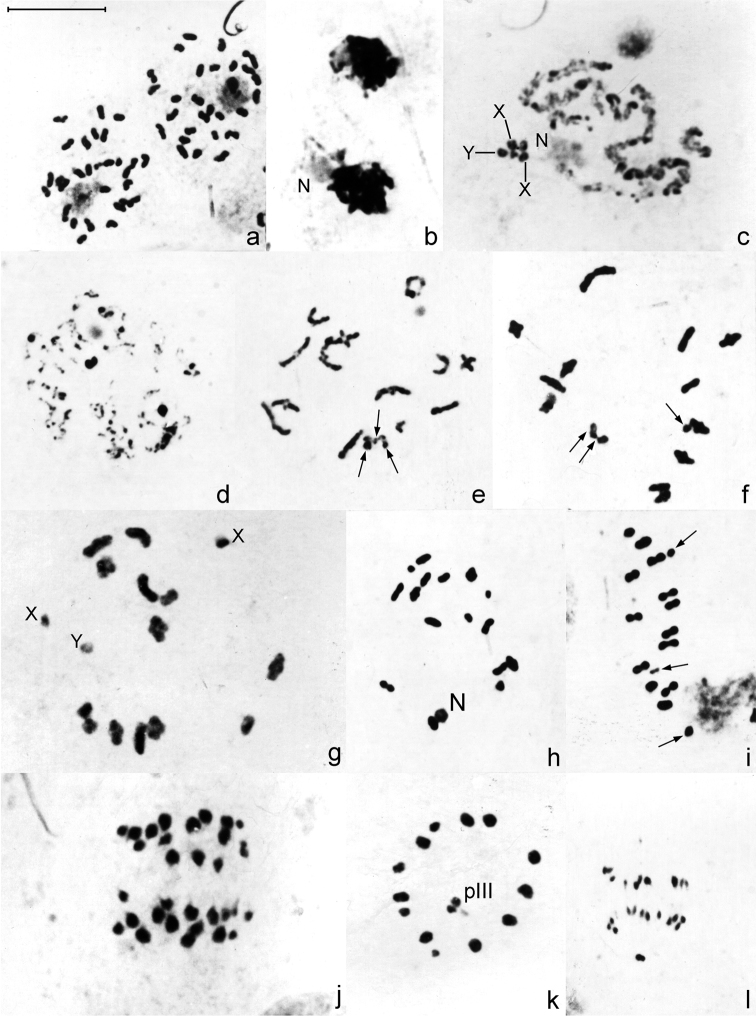
Male meiosis in *Belostoma elongatum* (**b, c, g, j, k**) and *Belostoma gestroi* (**a, d, e, f, h, i, l**) stained with 2% iron-propionic haematoxylin. **a** Spermatogonial prometaphase **b** Synizesis **c** Pachytene, X and Y = sex chromosomes **d** Diffuse stage **e–f** Early diakinesis **g–h** Diakinesis **i** Metaphase I **j** Anaphase I **k** Metaphase II, Y sex chromosome is negatively heteropycnotic **l** Anaphase II. Arrows indicate sex chromosomes. pIII = pseudo-trivalent. N = nucleolus. Bar = 10 μm.

Analysis of spermatogonial prometaphase of *Belostoma elongatum* and *Belostoma gestroi* revealed a diploid number of 29 chromosomes; both karyotypes were as described by Papeschi (1992) (Fig. 2a). Male meiotic behaviour in *Belostoma elongatum* and *Belostoma gestroi* was similar and followed the same pattern as previously described for other *Belostoma* species. Thus, we showed a single and combined Figure 2 with meiotic stages from both species. At synizesis, the first meiotic identifiable stage of meiosis, chromatin condenses eccentrically in the nucleus (Fig. 2b). At pachytene, an autosomal bivalent is associated with the nucleolus and the 13 autosomal bivalents are joined through their positive heteropycnotic terminal regions. The condensed sex chromosomes, close to each other, may be distinguished (Fig. 2c). In this cell, the two X chromosomes have a secondary constriction, but these constrictions are observed in only one specimen of *Belostoma elongatum*. During the diffuse stage, all bivalents decondense completely, except for some chromocentres (Fig. 2d). In *Belostoma gestroi*, at early diakinesis, both X chromosomes are negative heteropycnotic, and the Y chromosome is positive heteropycnotic (Fig. 2e, f). At late diakinesis, the three sex univalents and the 13 autosomal bivalents becomes isopycnotic in both species (Fig. 2g, h). Each bivalent has a single chiasma in either subterminal or terminal positions (Fig. 2e–h). At metaphase I, autosomal bivalents arrange in a ring, but the Xs and Y univalents do not show a defined position (Fig. 2i). During anaphase I, the bivalents divide reductionally, whereas the sex chromosomes do so equationally (Fig. 2j). All telophase I nuclei exhibit 16 chromosomes in each pole (13 + X_1_X_2_Y). The second meiotic division follows without an interkinesis stage. At metaphase II, the 13 autosomes dispose forming a ring and in the centre of it, the sex chromosomes are associated in a pseudo-trivalent. The Y chromosome is negatively heteropycnotic and is oriented towards the opposite spindle pole to that of X_1_ and X_2_ (Fig. 2k). At anaphase II, 14 chromosomes migrate to one pole (13 + Y) and 15 to the opposite one (13 + X_1_X_2_) (Fig. 2l).

### Chromosome bandings

**Figure 3.**
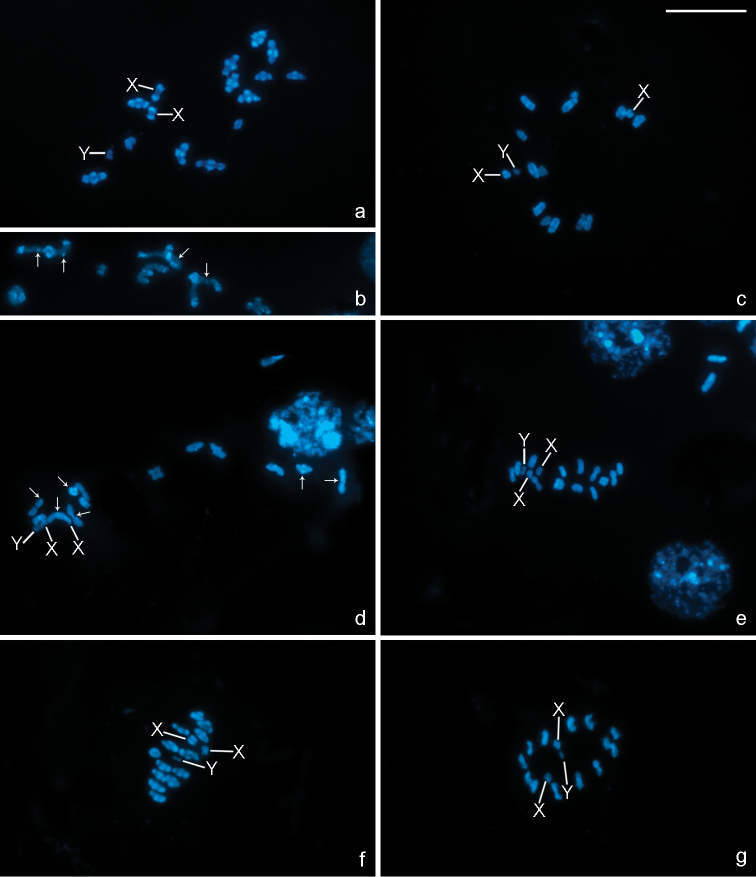
C-banding in chromosomes of *Belostoma elongatum* (**a–c**), *Belostoma gestroi* (**d, e**) and *Belostoma dentatum* (**f, g**) stained with DAPI. **a** Diakinesis, conspicuous terminal C-positive blocks are observed in all autosomal bivalents and both X chromosomes **b** A detail of autosomal bivalents with interstitial C-positive dots (arrows) at early diakinesis **c** Late diakinesis **d** Diakinesis, small terminal C-positive bands in some autosomal bivalents (arrows) **e** Metaphase I **f** Late diakinesis, terminal C-positive bands in all autosomal bivalents and both X chromosomes **g** Metaphase II. **a, c–g** The Y chromosome is C-negative. X, Y = sex chromosomes. Bar = 10 μm.

C-banding reveals differences in the amount and location of heterochromatin among the three species analysed. In *Belostoma elongatum*, very large C-positive blocks can be detected terminally on all bivalents from prophase I to metaphase I, and interstitial dots are also observed (Fig. 3a–c). In *Belostoma gestroi*, in contrast, C-positive bands are very small and are always located terminally (Fig. 3d, e). The results observed in *Belostoma dentatum* matched data previously described by Papeschi (1991) with C-positive bands terminally located in all bivalents (Fig. 3f, g). Furthermore, the two X chromosomes in the three species show terminally located bands, whereas the Y chromosome is C-negative (Fig. 3a, c–g).

**Figure 4.**
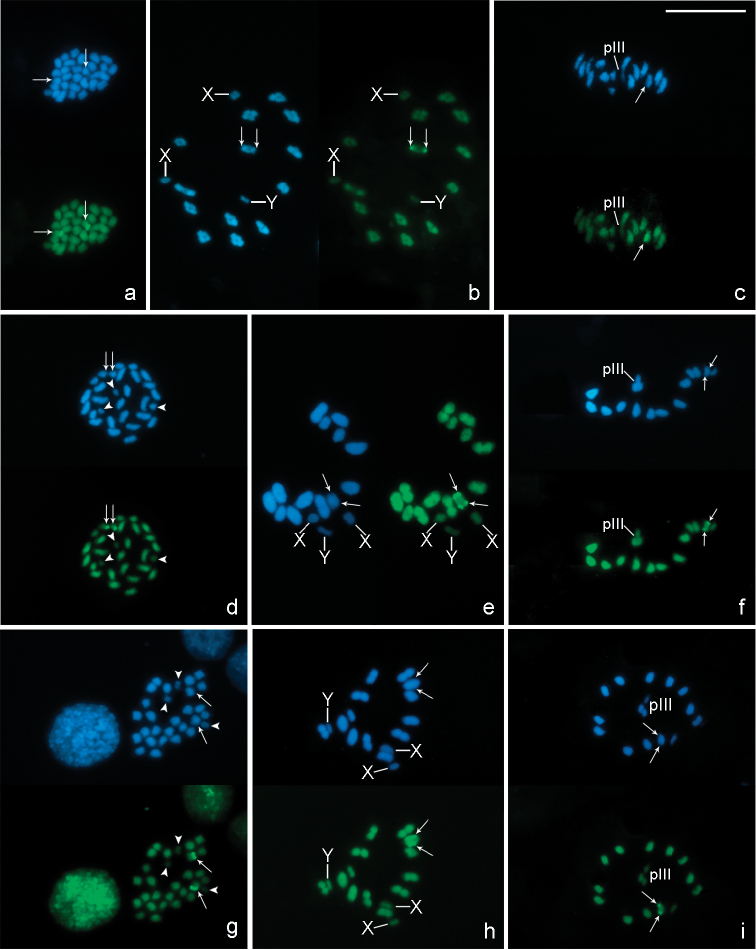
DAPI (blue) and CMA_3_ (green) fluorescent banding in chromosomes of *Belostoma dentatum* (**a–c**), *Belostoma elongatum* (**d–f**) and *Belostoma gestroi* (**g–i**). **a** Oogonial metaphase (2n = 30 = 26 + X_1_X_1_X_2_X_2_) **b** Diakinesis **c** Metaphase II **d** Spermatogonial metaphase (2n = 29 = 26 + X_1_X_2_Y) **e** Diakinesis **f** Metaphase II **g** Spermatogonial metaphase (2n = 29 = 26 + X_1_X_2_Y) **h** Diakinesis **i** Metaphase II. Arrows indicate DAPI negative/CMA_3_ positive bands. Arrowheads show sex chromosomes (**d, g**). X, Y = sex chromosomes. pIII = pseudo-trivalent. Bar = 10 µm.

All chromosomes stain homogenously with both fluorochromes on mitotic and meiotic metaphase cells in the three species, except for one of the medium-sized autosomal bivalents in *Belostoma dentatum* (Fig. 4a–c) and *Belostoma elongatum* (Fig. 4d–f), and one of the large-sized in *Belostoma gestroi* (Fig. 4g–i), which show a DAPI negative/CMA_3_ positive band at one terminal position.

### Location of rDNA

**Figure 5.**
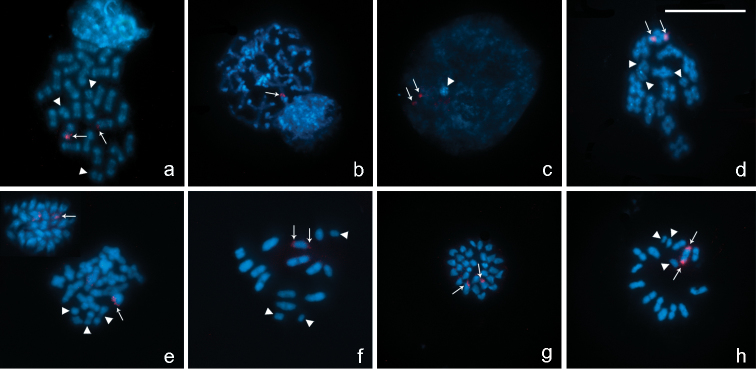
Location of rDNA genes in chromosomes of *Belostoma dentatum* (**a–d**), *Belostoma elongatum* (**e, f**) and *Belostoma gestroi* (**g, h**) by FISH with 18S rDNA probes (red signals, arrows). Chromosomes were counterstained with DAPI (blue). **a** Spermatogonial anaphase (2n = 29 = 26 + X_1_X_2_Y) **b** Pachytene **c** Diffuse stage **d** Diakinesis **e** Spermatogonial metaphase and diakinesis **f** Metaphase I **g** Spermatogonial metaphase (2n = 29 = 26 + X_1_X_2_Y) **h** Diakinesis-Metaphase I. Arrowheads show sex chromosomes. Bar = 10 µm.

In chromosome preparations of *Belostoma dentatum*, FISH experiments with the 18S rDNA probe show a cluster of rDNA genes located at one end of two homologous chromosomes each (Fig. 5a). A single cluster of signals is observed in an autosomal bivalent at pachytene (Fig. 5b). During diffuse stage, hybridization signals are observed in the decondensed mass of autosomal chromatin, whereas the sex chromosomes remain condensed forming a conspicuous DAPI bright chromatin body without any signals (Fig. 5c).  At diakinesis-metaphase I, one medium-sized autosomal bivalent show hybridization signals at both ends (Fig. 5d). In concordance with the results of *Belostoma dentatum*, in mitotic metaphases of *Belostoma elongatum* and *Belostoma gestroi*, hybridization signals are detected in two homologous autosomes (Fig. 5e, g). At diakinesis-metaphase I, a single cluster of rRNA genes is located at both ends of a medium-sized autosomal bivalent of *Belostoma elongatum* (Fig. 5e–f) and of a one large-sized of *Belostoma gestroi* (Fig. 5h).

## Discussion

The *Belostoma* species analyzed here shared apparently similar karyotypes, since they possess the same chromosome complement (2n = 29 = 26 + X_1_X_2_Y, male), with chromosomes progressively decreasing in size. In Belostomatidae, this 2n is the modal diploid chromosome number and is present in 10 species of *Belostoma* (Papeschi and Bressa 2006, Chirino and Bressa 2011). On the other hand, in *Belostoma elongatum* and *Belostoma gestroi* the male meiotic behaviour followed a similar pattern as previously described for other species of this genus (Papeschi and Bidau 1985, Papeschi 1996, Bardella et al. 2012). Both *Belostoma elongatum* and *Belostoma gestroi*, as well as *Belostoma dentatum*, showed the following cytogenetic characteristics: a) synizesis observed, b) a multiple sex chromosome system (X_1_X_2_Y, male), c) Y chromosome negatively heteropycnotic at metaphase II, d) variation in the TCL and in the C-banding pattern, and e) a single pair of NOR-autosomes. Within Heteroptera, the synizesis stage was described in a few species of *Belostoma* (Papeschi and Bidau 1985, Papeschi 1992) and *Dysdercus* Guérin-Méneville, 1831 (Bressa 2003, Bressa et al. 2003). In this stage the chromatin condenses eccentrically in the nucleus and chromosome pairing begins.

Heterochromatin characterization in the three species revealed differences in the amount, distribution and location of the constitutive heterochromatin in autosomes and both X chromosomes: i) terminal C-positive bands in *Belostoma dentatum*, ii) conspicuous C-positive bands at terminal and interstitial positions in *Belostoma elongatum*, and iii) very scarce C-positive bands terminally located in *Belostoma gestroi*. This variation in the constitutive heterochromatin of these three species could imply changes in the DNA content in the karyotype evolution in the genus, which could modify the size of the chromosome complement. In accordance with this suggestion, the analysis of TCL showed a significant variation among the three species, which means that certain chromosomal changes, must have taken place during their evolution.

**Figure 6.**
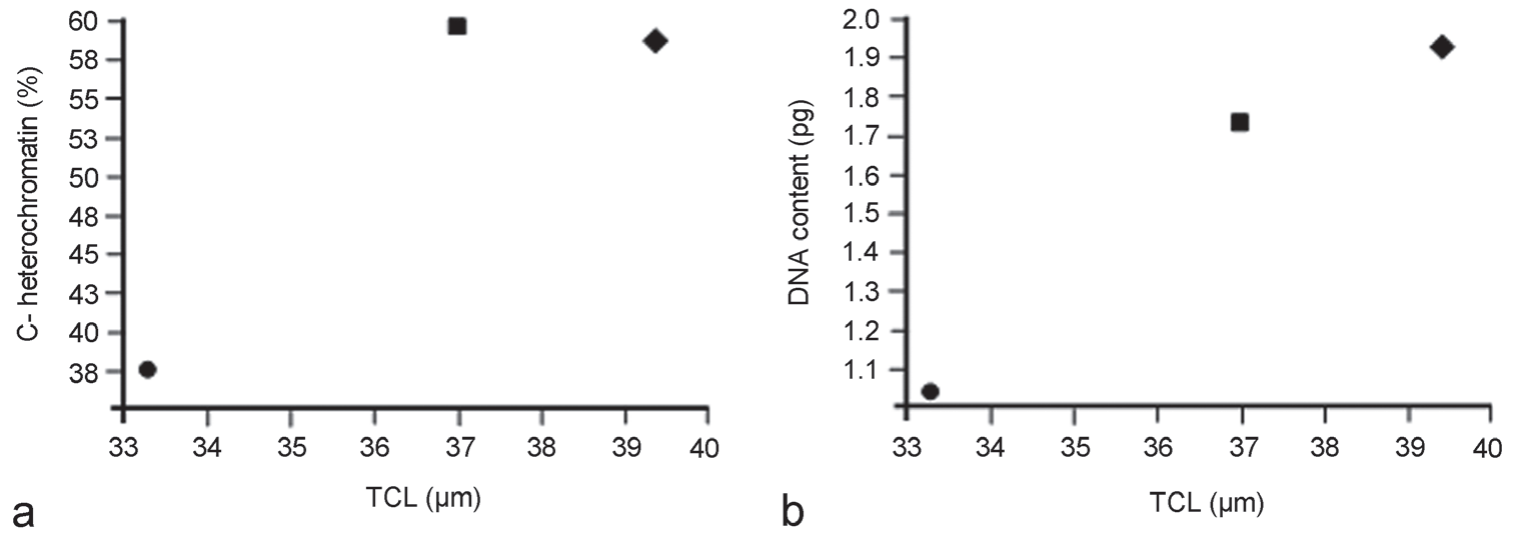
**a** Comparison between the total chromosome length (TCL) and the percentage of C-positive heterochromatin at diakinesis in *Belostoma dentatum* (58.54 ± 1.27 %; circle), *Belostoma elongatum* (59.47 ± 0.78 %; rectangle) and *Belostoma gestroi* (37.24 ± 1.50 %; diamond) **b** Comparison between the total chromosome length (TCL) and the haploid DNA content (pg) in *Belostoma dentatum* (1.93 ± 0.16 μm; circle), *Belostoma elongatum* (1.75 ± 0.05 μm; rectangle) and *Belostoma gestroi* (1.13 ± 0.13 μm; diamond). Data of percentage of C-positive heterochromatin and the haploid DNA content were obtained from Papeschi (1991, 1992).

Papeschi (1992) found a great interspecific variation in DNA content as well as differences in C-positive heterochromatin percentage among *Belostoma dentatum* (1.93 pg, 58.54 %), *Belostoma elongatum* (1.75 pg, 59.47 %) and *Belostoma gestroi* (1.13 pg, 37.24 %). Taking into account the data previously described by Papeschi (1992), together with the results obtained from the analysis of the TCL in these three species, we propose the existence of positive relationships between TCL and DNA content and between TCL and C-positive heterochromatin percentage (Fig. 6). Thus, it is apparent that differences in the TCL in the three species might represent changes or variations in DNA content since the accumulation/addition of satellite DNA in all chromosomes of the complement. The comparison between the DNA content and the C-positive heterochromatin percentage of the three species shows a general trend, i.e. an increase in the DNA content is accompanied by an increase in the amount of C-positive heterochromatin. However, *Belostoma dentatum* and *Belostoma elongatum* have a very different DNA content and a similar percentage of C-positive heterochromatin. On the other hand, *Belostoma gestroi* shows the lowest DNA content and the lowest C-positive heterochromatin percentage. In accordance with the earlier reports on six other species of *Belostoma* (Papeschi and Bidau 1985, Papeschi 1988, 1991, 1992), the genome size differences between *Belostoma dentatum* and *Belostoma elongatum* could be due to a proportionate variation of both C-positive heterochromatin and C-negative chromatin occurred during evolution. The chromosomes of *Belostoma gestroi* could have gained low amount of C-positive heterochromatin, or else during evolution some C-positive bands became lost.

In Heteroptera, the classical distribution pattern of C-positive heterochromatin is terminal in some or all chromosomes. Interstitial C-positive bands are described in a few species and some of them correspond to secondary constrictions and NORs. In concordance with these cytogenetic features, the C-banding pattern observed in *Belostoma elongatum* with respect to both terminal and interstitial C-positive regions agrees with most previous reports within *Belostoma* (Papeschi 1995) and Heteroptera (Camacho et al. 1985, Panzera et al. 1995, Grozeva and Nokkala 2001, Angus et al. 2004, Ituarte and Papeschi 2004, Bressa et al. 2005, 2008, Franco et al. 2006).

The results with fluorescent banding indicate that all C-positive bands in the species analysed were not enriched with AT or CG base pairs, as all chromosomes were stained homogeneously with both DAPI and CMA_3_ fluorochromes, except for the C- positive band observed in the medium-sized autosomal bivalent of *Belostoma dentatum* and *Belostoma elongatum* and in one of the large-sized of *Belostoma gestroi*, which was DAPI negative/CMA_3_ positive. Therefore, the CMA_3_ bright band is enriched in GC base pairs and could represent an NOR (see below). The presence of a CMA_3_ bright band was also reported not only in other species of *Belostoma* (Papeschi and Bressa 2006) but also in other heteropteran species, at interstitial or terminal position, either on autosomes or sex chromosomes, and they are generally associated to NORs (González-García et al. 1996, Papeschi et al. 2001, 2003, Rebagliati et al. 2003, Cattani et al. 2004, Grozeva et al. 2004, Poggio et al. 2011).

In Belostomatidae, the location of NORs was previously analysed by FISH with 18S rDNA probe in *Belostoma oxyurum* (Dufour, 1863) (2n = 6 + XY, NOR in sex chromosomes), *Belostoma micantulum* (Stål, 1860) (2n = 14 + XY, NOR in sex chromosomes), *Belostoma elegans* (Mayr, 1871) (2n = 26 + X_1_X_2_Y, NOR in a pair of autosomes) (Papeschi and Bressa 2006), and *Lethocerus patruelis* (Stål, 1854) (2n = 22 + 2m + XY, NOR in sex chromosomes) (Kuznetsova et al. 2012). The present paper presents information about the number and chromosomal location of ribosomal clusters in *Belostoma dentatum*, *Belostoma elongatum* and *Belostoma gestroi*, which have a single cluster located in an autosomal pair. In these three species the NOR is associated with a CMA_3_-bright band. The results of the fluorescent banding and FISH in these species agree with those described for *Belostoma oxyurum*, *Belostoma micantulum* and *Belostoma elegans*, in which the NOR regions are colocalized with a CMA_3_-positive band and, therefore, the repeating unit of rDNA is GC-rich (Papeschi and Bressa 2006). Taking into account the data on the number and location of rDNA clusters along with the type of sex chromosome systems in Belostomatidae, we can observe two different patterns of rDNA distribution. The NOR is located at terminal position on both sex chromosomes in species that have a simple sex chromosome system (XY), or, in contrast, the NOR is placed at terminal position on an autosomal pair in the species with a multiple sex chromosome system (X_1_X_2_Y). Hence, our present results led us to propose that in Belostomatidae the location of rDNA genes could be associated with variants of the sex chromosome systems. Moreover, this relationship between the number and location of the NOR and the sex chromosome system has only been observed in this family of Heteroptera.

Previous cytogenetic data on worldwide Belostomatidae species allowed Papeschi and Bressa (2006) to propose an ancestral male karyotype 2n = 28 = 26 + XY, from which the karyotypes with multiple sex chromosome systems (2n = 26 + X_1_X_2_Y) and those with a low 2n (6 + XY, 14 + XY, 13 + XY, 22 + XY) would have originated by fragmentation of the ancestral X chromosome and chromosomal fusions, respectively. It is generally accepted that multiple sex chromosome systems in Heteroptera are the result of fragmentation (s) of the X and/or Y chromosome (s) of an ancestral simple sex chromosome system (Heizer 1950, Hughes-Schrader and Schrader 1961, Ueshima 1979, Manna 1984, Papeschi 1996, Papeschi and Bressa 2006). The holokinetic nature of heteropteran chromosomes and the achiasmatic male meiosis of sex chromosomes are the main facts that support this hypothesis and may account for the variability (Ueshima 1979, Manna 1984, Thomas 1987). In most cases of multiple sex chromosomes, the increase in the number of sex chromosomes is not accompanied by a reduction in the number of autosomes. The analysis of different populations of *Belostoma orbiculatum* Estévez and Polhemus, 2001 (Papeschi 1996), *Belostoma plebejum* (Stål, 1858) (Papeschi 1994), *Belostoma dilatatum* (Dufour, 1863) (Bardella et. al 2012), *Oechalia pacifica* (Stål, 1859) (Heizer 1950) and *Banasa zeteki* Sailer, 1959 (Pentatomidae) (Schrader and Hughes-Schrader 1958), polymorphic for the sex chromosome systems, lend support to the hypothesis of a fragmentation origin of a multiple sex chromosome systems. In all these examples one chromosome of the simple system (XY) was replaced by two chromosomes of smaller size in the mutant individuals. Therefore, these interspecific polymorphisms of sex chromosomes represent a direct evidence of the origin of multiple sex chromosome system through fragmentation in *Belostoma*. On the other hand, the species of this genus with reduced chromosome numbers are characterized by a larger chromosome size, a low DNA content and very scarce C-positive heterochromatin (Papeschi and Bressa 2006). These karyotypes probably originated from the ancestral chromosome complement through chromosome fusions. The possibility of their occurrence is supported by the fact that the autosomal fusions have been found in heterozygous condition in natural populations of *Belostoma plebejum* (Papeschi 1994), *Triatoma infestans* (Klug, 1834) (Poggio et al. 2013) and *Mepraia gajardoi* Frías, Henry and González, 1998 (Pérez et al. 2004) (Reduviidae).

**Figure 7.**
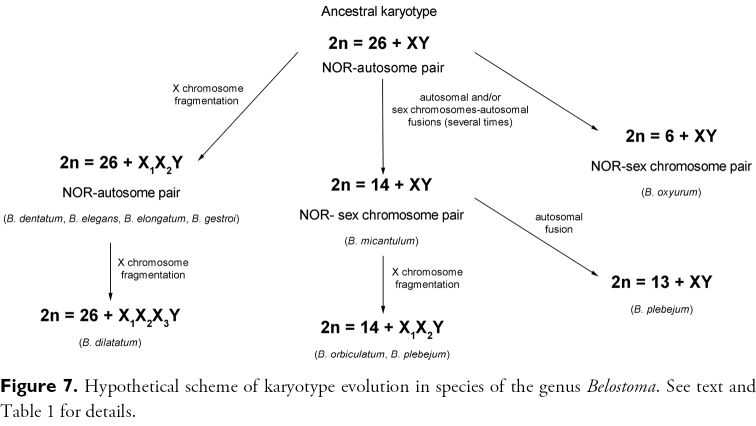
Hypothetical scheme of karyotype evolution in species of the genus *Belostoma*. See text and Table 1 for details.

Published data on karyotype evolution in species of this genus (Papeschi and Bressa 2006) along with the present results of rDNA-FISH support the hypothesis that in the ancestral male karyotype (2n = 28 = 26 + XY) the NOR would have been located in a pair of autosomes (Fig. 7). A fragmentation of the single X chromosome in the ancestral karyotype resulted in multiple X chromosomes and led to a karyotype with 2n = 29 = 26 + X_1_X_2_Y, but keeping the ancestral NOR-autosome pair, as represented by *Belostoma dentatum*, *Belostoma elegans*, *Belostoma elongatum* and *Belostoma gestroi* (Fig. 7). On the other hand, autosomal fusions and the fusion of the ancestral sex chromosome pair (XY) with the autosomes carrying the NOR would result in the reduction in the diploid chromosome (2n = 8, 16), increasing the chromosomes size and led to the presence of rDNA genes in both X and Y chromosomes. Alternatively, the rDNA gene cluster could have been translocated from an autosomal location to the X and Y chromosomes, as it has been proposed in *Dysdercus albofasciatus* Berg, 1878 (Bressa et al. 2009). Furthermore, future studies within *Belostoma* about this regular pattern will shed light on the karyotype evolution within the genus and also support the mechanisms involved in the karyotype evolution.

Conventional taxonomy of water bugs has focused almost entirely on adult specimens. There are relatively few publications on interspecific differences among the larvae, and fewer still concern South American species. The literature of the genus *Belostoma* includes much confusion because, in many cases, the species are very similar in coloration and appearance and only males or rarely only females can be identified (Heckman 2011). In *Belostoma dentatum*, *Belostoma elongatum* and *Belostoma gestroi*, the karyotype analyses allow us to get a detailed characterization and a better knowledge of their chromosomal structure. Hence, we conclude that the cytogenetic studies provide valuable features that can be used to solve problems on taxonomic identification, at least for this genus.
